# Diverting the Use of Hand-Operated Tablet Press Machines to Bioassays: A Novel Protocol to Test ‘Waste’ Insoluble Shell Matrices

**DOI:** 10.3390/mps7020030

**Published:** 2024-04-01

**Authors:** Camille Lutet-Toti, Marie Da Silva Feliciano, Nelly Debrosse, Jérôme Thomas, Laurent Plasseraud, Frédéric Marin

**Affiliations:** 1 UMR CNRS-uB-EPHE 6282 ‘Biogéosciences’, Université de Bourgogne, 21000 Dijon, France; 2 Dipartimento di Chimica “Giacomo Ciamician”, Alma Mater Studiorum Università di Bologna, 40126 Bologna, Italy; 3 Institut de Chimie Moléculaire de l’Université de Bourgogne, ICMUB UMR CNRS-uB 6302, 21000 Dijon, France; laurent.plasseraud@u-bourgogne.fr

**Keywords:** shell extract, recycling, bioactive factors, insoluble matrix, hand-operated tablet press machine

## Abstract

To mineralize their shells, molluscs secrete a complex cocktail of proteins—collectively defined as the calcifying shell matrix—that remains occluded in the exoskeleton. Nowadays, protein extracts from shells are recognized as a potential source of bioactive substances, among which signalling molecules, bactericides or protease inhibitors offer the most tangible perspectives in applied sciences, health, and aquaculture. However, one technical obstacle in testing the activity of shell extracts lies in their high insolubility. In this paper, we present a protocol that circumvents this impediment. After an adapted shell protein extraction and the production of two organic fractions—one soluble, one insoluble—we employ a hand-operated tablet press machine to generate well-calibrated tablets composed of 100% insoluble shell matrix. FT-IR monitoring of the quality of the tablets shows that the pressure used in the press machine does not impair the molecular properties of the insoluble extracts. The produced tablets can be directly tested in different biological assays, such as the bactericidal inhibition zone assay in Petri dish, as illustrated here. Diverting the use of the hand-operated tablet press opens new perspectives in the analysis of insoluble shell matrices, for discovering novel bioactive components.

## 1. Introduction

In Europe, the consumption of shellfish of economic interest, such as oysters, mussels, clams, scallops, or cockles generates huge amounts of waste in the form of empty shells [1]. The partial recycling of this mass-produced by-product consists mostly in applications of low-added value: constituents of concretes [2], dietary supplement for poultry [3], amendment of acidic or heavy metal-contaminated soils [4], and depollution of industrial waters [5]. Yet, shells are not only 100% mineral objects. They are biocomposite materials consisting of the superimposition of two to three calcified layers exhibiting different microstructures, each of them containing about 0.1 to 2% organics, collectively defined as the shell matrix [6]: indeed, when they mineralize their shells, molluscs—through their specialized organ, the mantle—secrete a complex cocktail of proteins and saccharides that interact with the inorganic mineral precursors—calcium and bicarbonate—and remain occluded in the mineral phase [6]. This shell matrix is usually retrieved by dissolving the mineral phase. Over decades, thousands of fundamental studies have biochemically characterized the shell matrix, in particular the protein moieties. While early works from the seventies/eighties evidenced the pivotal role of shell proteins in regulating mineral deposition [7,8], only recently was it discovered that this mixture may display numerous additional molecular and cellular key functions in connection with biomineralization [9].

Indeed, recent high-throughput screening, combining transcriptomics on mollusc mantle and proteomics on shell extracts, has shown that the shell matrix is composed of tens—not to say hundreds—of proteins that can be classified in several families [9,10,11]. While many proteins, in particular those with low-complexity domains (LCDs) are orphans of functions, others exhibit amino acid sequences that bring them close to bioactive factors of interest, such as bactericides, signalling molecules or protease inhibitors [12,13]. Today, shell proteins, and more generally shell macromolecules, are considered as a potential reservoir of useful substances, not only in biomaterials domain, but also in health research, aquaculture, and zootechnics [9,14].

One major drawback of shell protein extracts is that many of them are totally insoluble, and therefore not easily—not to say, not at all—testable for their biological properties. To give a striking example, the organic matrix of nacre—the most iconic and studied mollusc shell microstructure so far—represents between 1 and 2% of nacre weight, but 90% of this matrix is insoluble [15] in standard aqueous decalcifying solutions (acids, or EDTA), and consequently, this fraction is ignored for further analysis of its in vitro bioactivity. In other words, only 10% of nacre matrix, corresponding to the most soluble moieties, is currently used for testing its effects in the presence of cells [16,17].

In this paper, we present a protocol that circumvents this drawback. We have developed a mild shell matrix extraction that identifies two fractions: an acetic acid-soluble one and an acetic acid-insoluble one. While the soluble fraction can be further subdivided into smaller fractions according to different ultrafiltration cutoffs, the insoluble fraction, after lyophilization, can be transformed in tablets, owing to a hand-operated tablet press machine, and the resulting tablets can be tested in vitro, in Petri dishes, for their effects in the presence of cells, here, a marine pathogenic vibrio strain. FT-IR monitoring shows that the pressure used for making tablets does not affect the chemical functions of the insoluble matrix. We suppose that our protocol is extremely useful for testing in bulk all shell matrix components—and not only soluble ones—in order to discover novel biological properties of interest in aquaculture and health.

## 2. Materials and Methods

### 2.1. Material Samples

The four species selected for the study are all commercially available; they live along the coasts of Western Europe. As bivalves, they are not subject to the European Directive 2010/63/UE relating to the protection of animals used for scientific purposes. The shells were collected from fresh living animals in a local supermarket in Quetigny, Burgundy, France. They include the Pacific edible oyster (*Magallana gigas*, formerly *Crassostrea gigas*), the warty venus (*Venus verrucosa*), the Manila clam (*Venerupis philippinarum*), and the common cockle (*Cerastoderma edule*).

Shells were carefully sorted visually and specimens with no shell defects, and no epibionts were chosen. The selected shells were opened with a knife and the soft tissues were scrupulously removed. In particular, the junctions between pallial muscles and shells were carefully scrubbed, as well as the outer shell surfaces. The leathery hinges were removed with a knife and the shells were incubated at room temperature in an aqueous solution of sodium hypochlorite (0.26% active chlorine, 10 times dilution) for two days, on a laboratory rotary shaker, with at least four changes in solution. Shells were then extensively rinsed with tap water and dried. Dried shells were mechanically cleaned: in particular, the hinges were cut from the shells with a diamond saw (Dremel rotary saw). In addition, all remains of epibionts and periostracum (if still present) were removed by abrasion with a dental drill. Shells were cleaned again with dilute sodium hypochlorite (0.26% active chlorine) for hours then thoroughly rinsed with double distilled water and dried at 40 °C. Shells were coarsely crushed in a mechanical jaw-crusher (BB200 model, Retsch, Eragny, Luxemburg), and fragments were further milled with an agate mortar grinder (Pulverisette 2 model, Fritsch, Idar-Oberstein, Germany) and the resulting powder was sieved to 200 µm. The powders were stored in dry containers for further use.

### 2.2. Shell Matrix Extraction

For each species, the powder (25 g) was resuspended in double-distilled water (DDW, around 20 mL) in a glass beaker and decalcified overnight by addition of 200 µL of 10% (vol/vol) glacial acetic acid, every 5 s, to reach a final volume of 1 L. The solution was constantly stirred with a magnetic glass bar. The resulting clear solution was centrifuged at 3893× *g* for 20 min. The pellet, containing the acid-insoluble matrix (AIM), was collected and resuspended in DDW before being centrifuged (10 min, 3893× *g*). Five cycles of resuspension–centrifugation were performed to ensure the removal of all remaining salts, acetic acid, and soluble matrix. One drop of the 5th supernatant was put on pH indicator paper to check that the solution was around neutrality. All the intermediate supernatants were added to the initial supernatant. The collected AIM was then lyophilised in a freeze-dryer (Telstar Cryodos, Terrassa, Spain) after a preliminary quick-freeze in liquid nitrogen.

The supernatant, defined as the acid-soluble matrix (ASM), was further filtered on a 5 µm membrane mounted on a Nalgene filtration device connected to a pump (Millipore France, Molsheim). The ASM was then ultrafiltered in an ultrafiltration stirred cell (Millipore, model 8400, 400 mL) against a membrane of 10 kiloDalton (kDa) cutoff. Both retentate (>10 kDa) and filtrate (<10 kDa) were collected, and the filtrate was ultrafiltered again on a 1 kDa cutoff membrane. The new retentate (>1 kDa and <10 kDa) was collected. In one case (*Venerupis philippinarum*), since the 10 kDa filtrate was too viscous, i.e., generating a low flow rate, an intermediate ultrafiltration cutoff of 3 kDa was added. All soluble fractions (>1 kDa) were dialyzed against ultra-pure water, with a minimum of 5 water changes (over two days), in a ready-to-use dialysis bag (SpectraP/Por 6 dialysis membrane, pre-wetted RC tubing) of 1 kDa cutoff.

All soluble matrices including fractions of ASM > 10 kDa, of 1 < ASM < 10 kDa, and, for *V. philippinarum*, of 1 < ASM < 3 kDa and 3 < ASM < 10 kDa, were freeze-dried similarly to the AIMs. After complete lyophilisation, all fractions (AIMs and ASMs) were weighed on a precision balance (Quintix35-1S model, precision 0.01 mg, Sartorius, Göttingen, Germany) for their quantification.

### 2.3. Hand-Operated Tablet Press for AIMs

The freeze-dried AIM was transformed into pellets with a hand-operated tablet press (HP-mini, LC-Instru, Lisses, France) by using a 5 mm diameter piston mould set (Maassen Spektroskopie, Möglingen, Germany). Briefly, chips of freeze-dried AIM were manually disaggregated with a clean spatula and subsequently homogenized in an agate mortar before being introduced into the piston mould set. A pressure of one ton was applied to produce tablets of 2 to 5 mg, which were weighed on the same precision balance (as for lyophilisates) then stored individually, in dry conditions, in 24-well polystyrene flat bottom microplates (Nunc, Roskilde, Denmark) before use.

### 2.4. Fourier Transform Infra-Red Spectroscopy of AIMs

FT-IR spectroscopy was performed to monitor whether the applied pressure (1 ton) did not denature the extracts nor modify their chemical functions. To this end, for each species, FT-IR spectra were acquired on AIM samples that were not submitted to pelletisation and on tablet-formed AIMs. An FT-IR Bruker Alpha spectrometer was used (ICMUB, Dijon, France): this apparatus was equipped with an Attenuated Total Reflectance (ATR) ALPHA-P device comprising a mono-reflection diamond crystal. Spectra were recorded in a 4000–375 cm^−1^ wavenumber range, with 24 scans at a spectral resolution of 4 cm^−1^. The qualitative assignment of absorption bands was performed by comparison with previous spectra descriptions, achieved by our group or available in the literature [12,18]. We made an ultimate check on the potential effect of a high pressure on the FTIR spectra by making tablets with a hand-operated tablet press at high pressure (9 tons, 5 min, Atlas press, Specac, Orpington, UK) in the presence of potassium bromide (0.5% AIM in KBr) and few spectra were acquired in transmittance mode.

### 2.5. Antibacterial Assay

The produced AIM pellets were unconventionally used in disk diffusion assays, also called radial diffusion assay, adapted from the Kirby–Bauer Test [19] aiming at screening a large set of substances for their putative antibacterial properties. The test was performed on standard Petri dishes (94 mm diameter, Greiner Bio-One, Courtaboeuf, France). Before use, the pellets were sterilised under UV lights for 20 min on each side. *Aliivibrio salmonicida* (CIP103166T, Centre de Ressources Biologiques de l’Institut Pasteur, Paris, France), a marine fish pathogen, was grown in a liquid culture (marine broth 2216 DIFCO), lightly stirred for 11 days at 12 °C. Once in log phase, the culture suspension (600 µL) was spread using the “spread plate method” on marine agar (marine agar 2216 DIFCO). After absorption of the bacterial suspension by the medium, the sterile AIM pellets were evenly distributed on top of the agar. As a positive control, 7 mm diameter disks containing the Vibrio static agent O.129 (2,4-Diamino-6,7-diisopropylpteridine, ref. 53872, lot 64478673, 500 µg per disk, Bio-Rad, Hercules, CA, USA) were added to the test. The plates were allowed to incubate upside-down for 9 days at 12 °C. Inhibitions zones were observed around the pellets and their diameters were measured. Macrophotos were obtained with a Digital Nikon D750 Camera (Nikon, Tokyo, Japan), equipped with AF-S VR Micro-Nikkor 105 mm f/2.8 G IF-ED or AF-P DX Nikkor 18–55 mm f/3.5–5.6 G VR objectives. The test was reproduced in triplicate.

## 3. Results

### 3.1. Shell Matrix Extraction

**Figure 1:**
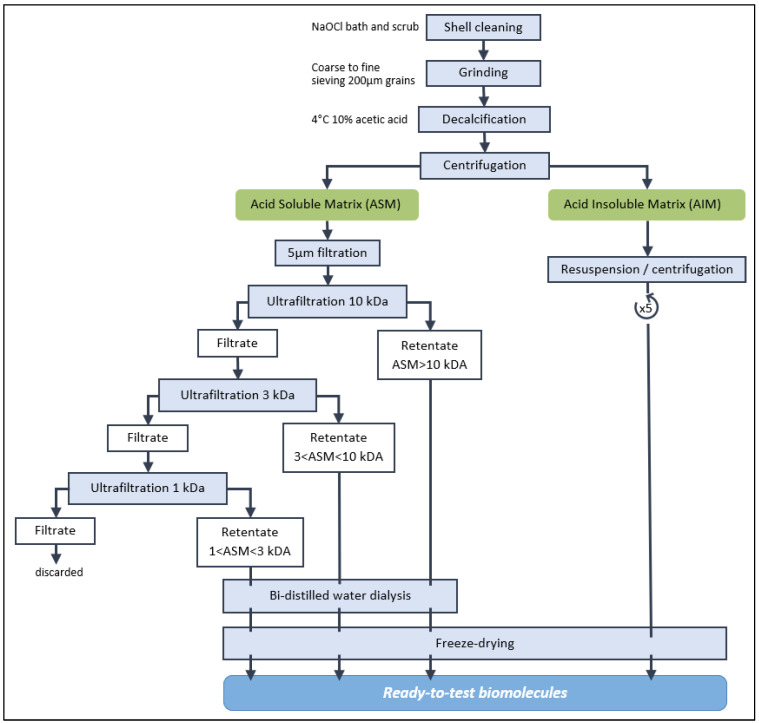
Flow chart of the extraction steps of shell matrices (acetic acid-soluble and insoluble). The ASM was passed in successive ultrafiltration filters of decreasing cutoff. The different ASMs and the AIM were *in fine* freeze-dried.

The flow chart of Figure 1 indicates the succession of steps that allow the obtaining of different organic fractions, depending, firstly, on their solubility (soluble vs. insoluble), and secondly—only for the soluble fraction—on their molecular weights when ultrafiltered on filters of different cutoffs (high-molecular-weight soluble fraction vs. low-molecular-weight soluble fraction). Two soluble fractions were obtained for *C. edule*, *M. gigas* and *V. verrucosa*, and three for *V. philippinarum*.

The quantification of the extracted shell matrices, obtained by weighing of the lyophilisates, is indicated in Table 1. The obtained matrix percentages vary between 0.08 (*C. edule*) and 0.55% (*M. gigas*) of the total shell powder. In other words, this represents 0.8 mg to 5.5 mg of matrix per gram of shell powder, respectively. Interestingly, the percentage of AIM compared with the total matrix (AIM + all ASM fractions) varies from 55% (*V. verrucosa*) to almost 78% (*V. philippinarum*). In all four examples, this represents the preponderant part of the total shell matrix.

### 3.2. Hand-Operated Tablet Press for Insoluble Matrix

**Figure 2:**
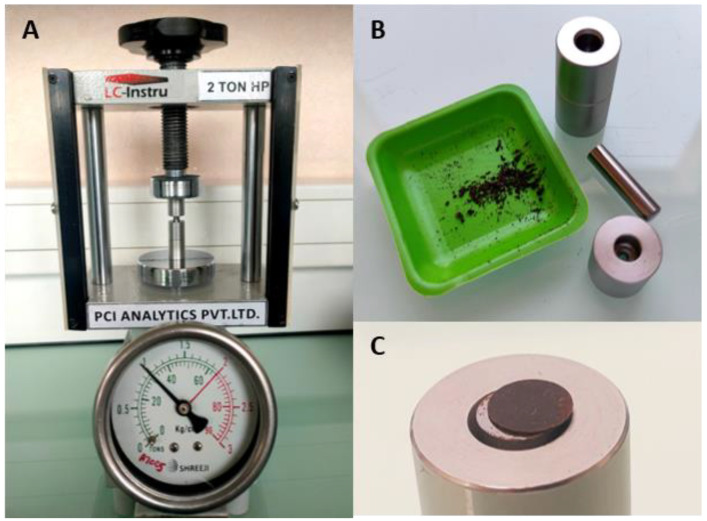
Pelletisation process. (**A**) Hand-operated tablet press machine, showing a force of 1 ton put onto the AIM to make a tablet (PH-mini, LC-Instru). (**B**) Freeze-dried AIM extracted from *Cerastoderma edule*, next to the pellet mould set. (**C**) Freshly produced AIM pellet of *C. edule*.

As shown in Figure 2, the use of a hand-operated tablet press machine generated tablets of AIM, of weights between 3.2 and 4.6 mg. The tablets were homogeneous, compact, and did not disaggregate. They could be handled easily with forceps and could be sterilized on each side by UV-light. Depending on the species considered, and taking into account the loss when manipulating the AIM lyophilisates, 100 g of initial shell powder allowed us to make between 13 (*C. edule*) and 123 (*M. gigas*) tablets.

### 3.3. Fourier Transform Infrared Spectroscopy

**Figure 3:**
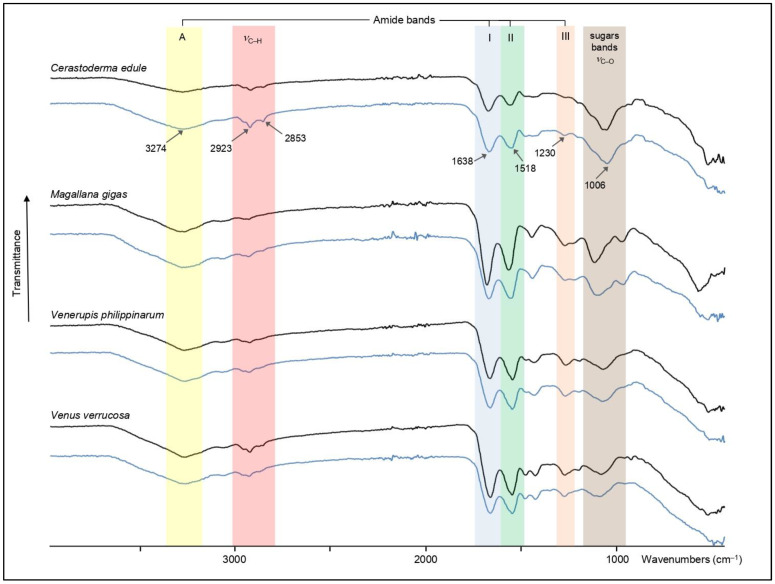
FT-IR spectra of the AIMs of the four tested bivalve shell matrices. The AIMs were monitored in two conditions: before (black curve) and after (blue curve) pelletisation. Note that the pelletisation process did not modify the IR signature of the spectra. A, I, II and III correspond to the different amide absorption bands of proteins.

Figure 3 illustrates the results obtained from AIM fractions of the four species. The four tested species exhibit an overall similarity of their IR spectral signature; i.e., most of the absorption bands are shared by all samples. The main differences are found in the 1700–400 cm^−1^ domain, where the relative amplitudes of the absorption bands can be rather dissimilar from species to species. Among the shared absorption bands, one finds the characteristic bands of proteins: the broad one between 3290 and 3260 cm^−1^ corresponding to amide A stretching (*ν*N–H), the amide I band (*ν*C=O stretching) between 1632 and 1648 cm^−1^, the amide II bands (*ν*C–N stretching) between 1534 and 1514 cm^−1^, and the amide III band (*ν*C–N stretching, *δ*NH bending) around 1226/1233 cm^−1^. In the latter case, we cannot discount that it combines to the *v*S=O vibration, frequently assigned to the presence of sulphate groups. The shoulders visible around 3500 cm^−1^, and more or less pronounced depending on the sample (in particular *Magallana* and *Venus*), suggest the presence of *v*OH absorptions. In addition to infrared bands of peptide linkage, all spectra present also *ν*C–O broad absorption bands specific to carbohydrates, between 1000 and 1080 cm^−1^ and the band around 2920–2930 cm^−1^, characteristic of (*ν*C–H) stretching vibrations. Other absorption bands of interest observed in all samples are located in the [1445–1390 cm^−1^] zone, which make a doublet in *Cerastoderma*, *Venerupis*, and *Venus*. One of these absorption bands is often attributed to carboxylate groups [*ν*_sym_(COO^−^)] [12].

Interestingly, when comparing the FT-IR spectra obtained from AIM samples not submitted to pelletisation to those after pelletisation, we observe that they are entirely superimposable, for each of the four species. This clearly suggests that the hand-operated tablet press treatment does not affect the chemical structure of the insoluble extracts.

As an ultimate check, we verified if a nine-times-higher pressure (9 tons, 5 min) than the one used for making our AIM tablets had an effect on the IR spectral properties of the extract. The control tablets, made with KBr and AIM, were read in transmittance mode. No modification of the spectra was recorded but was not shown here.

### 3.4. Antibacterial Assay on AIMs

**Figure 4:**
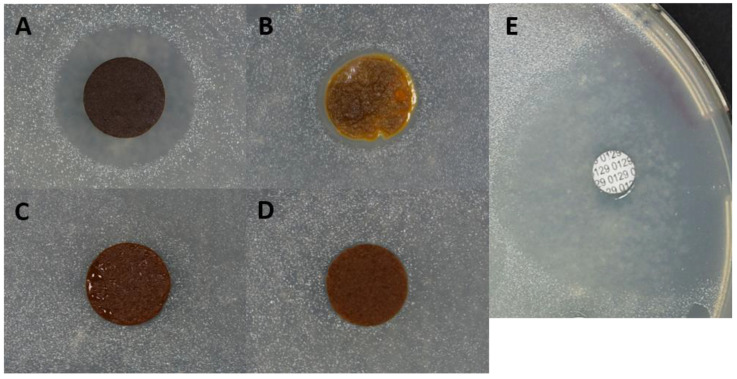
Antibacterial assays of AIM tablets on *Aliivibrio salmonicida* cultures grown on marine agar. (**A**) *Cerastoderma edule* AIM; (**B**) *Magallana gigas* AIM; (**C**) *Venerupis philippinarum* AIM; (**D**) *Venus verrucosa* AIM. (**E**) positive control with vibrio static agent O.129 (Bio-Rad). While (**C**,**D**) give negative signals, (**A**,**B**) induce inhibition zones, and show bactericidal ability against *A. salmonicida*. The positive control shows a large inhibition zone.

Figure 4 shows the effect of the four AIM tablets on the growth of *Aliivibrio salmonicida.* The *Cerastoderma edule* tablet (Figure 4A), as well as the *Magallana gigas* tablet (Figure 4B), exerts an inhibitory effect, since this bacterial strain does not grow in contact to the tablet; in other words, a clear growth inhibition zone is visible. The effect is stronger with *C. edule* AIM, and moderate for *M. gigas*. On the contrary, *Venerupis philippinarum* (Figure 4C) and *Venus verrucosa* (Figure 4D) do not exhibit any inhibitory effect. The control with Vibrio static agent O.129 shows a large inhibition zone. The test, performed in triplicate, gave consistent and reproducible results.

## 4. Discussion

In this paper we present a novel manner of utilizing insoluble matrices extracted from diverse mollusc shells of economic interest. From a theoretical viewpoint, our protocol, based on the complete decalcification of shell powder, allows the extraction of all the organics contained in shells, whatever they are (proteins, polysaccharides) and whatever their state in water or in dilute acid (soluble vs. insoluble). Interestingly, by using different ultrafiltration cutoffs, most of the acetic acid-soluble organic fractions (ASMs) are collected with a minimal loss and can be further analysed. The exception is the fraction inferior to 1 kDa, corresponding to very short peptides (fewer than ten amino acid residues on average) that pass through the 1 kDa ultrafiltration membrane: there is no easy way to concentrate this fraction and separate it from the free inorganic ions resulting from the decalcification, including calcium, carbonate, bicarbonate, and acetate. However, previous research suggests that the fraction below 1 kDa is extremely minor in comparison to the other soluble ones of a molecular mass above 1 kDa [20].

The main focus of this work is the acetic acid-insoluble fraction (AIM) and its subsequent use. Because of its high insolubility, the AIM is neither characterized biochemically in detail nor utilized in biological assays: most of the literature dealing with the bioactivity of shell extracts concerns only soluble fractions [16,17,21,22]. The peculiar insolubility property of AIM is known since the very first chemical analysis performed on shells (the ‘conchioline’ of Frémy) [23] and has been underlined many times [24]. Strong chaotropic solvents like guanidinium chloride, sodium dodecyl sulphate, or urea may help to solubilise the AIM, but its solubilisation is very partial; i.e., an important insoluble residue still remains after the treatment. Furthermore, these denaturing solvents should be proscribed when one wants to test the biological activity of the extracts.

From a cellular and molecular viewpoint, although poorly documented, it is admitted that the partial insolubilisation of the matrix occurs just after the secretion of water-soluble precursors in the space between the mantle and the shell mineralization front [25]. These soluble precursors are putatively crossed-linked/polymerized/sclerotized. Among the different molecular mechanisms involved, one may cite an oxidative process called ‘quinone-tanning’, which occurs on the DOPA-containing proteins [26]. Another mechanism involves the secretion of silk-fibroin-like proteins [27], forming, together with chitin, a gel that hardens by expelling water molecules and by making intermolecular cross-linking, to form chitin–protein complexes [24]. The end product, the AIM, is strong, flexible, and hydrophobic and is often organized in sheaths around crystallites. The presence of the sheaths, together with the ordered spatial organization of crystallites, precludes the propagation of cracks in the mineralized layer and contributes to reinforce the mechanical properties of the whole shell.

It is striking to observe that, in molluscs, the amount of shell matrix varies a lot, together with the ratio between soluble and insoluble fractions. For one given mollusc, these variations seem to depend on two parameters: its phylogenetic position in the mollusc tree and its shell microstructures. For example, most pteriomorphid bivalves are known to have shells with a higher organic matrix content (0.5% to more than 1%), while those of all caenogastropods are very poor in organics (between 0.1 and 0.01%) [28]. It is also known that shell microstructures [29] like calcitic prisms or nacre (both present in the edible mussel, or in the pearl oyster) contain a high proportion of matrix (usually more than 1% of the shell weight) and that this matrix is predominantly insoluble [15]. At the opposite end, other types of microstructures, such as some crossed-lamellar ones, are usually far less rich and exhibit mostly soluble components [28]. The four bivalves of commercial interest treated here represent one pteriomorphid (*M. gigas*) and three heterodonts (*C. edule*, *V. philippinarum*, *V. verrucosa*). We noticed a relatively high amount of organic matrix for the first one (0.55%, i.e., 5.5 mg of matrix per gram of shell powder), a species that exhibits a calcitic shell mostly composed of foliated and chalky microstructures, with a thin outer prismatic layer [29]. The amount of organics extracted from this species lies in the range indicated above [30].

The three heterodont bivalves (*V. verrucosa*, *V. philippinarum*, *C. edule*) exhibit lower amounts of organics (0.2% to less than 0.08%, meaning 2 to 0.8 mg of organics per gram of shell powder). These three specimens are entirely aragonitic and are composed of different combinations of composite prismatic, crossed-lamellar, complex crossed-lamellar, and homogeneous microstructures (Table 1). The amounts of organics extracted from these three species are congruent with earlier findings on heterodont bivalves [30]. Interestingly, we found out that, for the four species, the acetic acid-insoluble matrix (AIM) represents 55 to almost 78% of the total shell matrix, that is to say, quantitatively, the most important part of the matrix. In itself, this finding fully justifies focusing on this shell extract which is rarely—not to say never—utilized in bioactivity assays. In addition to this fact, proteomic investigations conducted by us [9,12,15] have shown that mollusc shell AIMs contain a complex cocktail of different proteins (up to few hundreds). Usually, for a given species, the AIM protein content partly overlaps with that of the corresponding ASM [31]. However, we also observed that AIMs contain usually more proteins than ASMs (F.M., in preparation). This constitutes another argument to find a manner to exploit in an optimized way the diversity of the protein content of shell AIMs.

To this end, we have diverted the use of a hand-operated tablet press machine to generate calibrated AIM tablets. This represents undoubtedly the best and easiest way to test shell AIMs in biological assays. Hand-operated tablet press machines are used normally for generating potassium bromide (KBr) tablets in standard FT-IR spectroscopy in transmittance mode, or for making drug tablets in pharmaceutical studies. At first sight, generating a high pressure (1 ton or more) for making tablets that can be easily manipulated with tweezers does not affect the AIM chemical properties, as its spectral IR signatures, checked and validated by FT-IR before and after pelletisation, did not show any variation and were fully superimposable. Our attempt to test the efficiency of the insoluble extracts in a hardly modified Kirby–Bauer test was successful, since two of the tested AIM tablets, that of *Cerastoderma edule* (the common cockle) and of *Magallana gigas* (the edible oyster), exhibited bactericidal capacity against the marine strain *Alivibrio salmonicida*, a pathogenic agent responsible for cold-water vibriosis in salmons. In consequence, our paper has demonstrated two points: (1) the feasibility of using shell AIM tablets in biological assays; (2) the capability of shell AIMs to induce different responses in biological assays. This clearly indicates that our ‘tablet’ approach is adapted to the screening of a large array of shell waste products from the shellfish industry, heliciculture, and pearl farming, in the perspective of discovering novel useful molecules of natural origin.

In conclusion, this paper emphasizes the utilisation of insoluble shell matrices, which are usually discarded in most applications so far. We are convinced that transforming ‘useless’ shell AIMs in tablets opens new possibilities for developing adapted bioactivity assays, such as the bactericidal assay illustrated here. Besides their relative abundance in many shells, AIM extracts are easier to produce than ASMs, since they require only the dissolution of the mineral phase, centrifugation, and thorough rinsing before freeze-drying. This line of research is one part of a general long-term strategy that follows the rules of green (sustainable) chemistry and aims at recycling and adding value to quantitatively abundant waste sea products, emptied shells from commonly consumed molluscs. In addition, our complete protocol, from the extraction to the production of tablets, employs standard, cheap, and highly accessible chemicals (sodium hypochlorite, acetic acid) and simple inexpensive processes (centrifugation, ultrafiltration).

## Figures and Tables

**Table 1:** Amounts of organic matrix in the shell of the four tested bivalves.

Mollusc Species		Mineralogy and Shell Microstructure *	Matrix Fraction	Mean Matrix Quantity (mg) per Gram of Shell Powder	% Matrix in Shell Powder	% AIM/Total Matrix
*Magallana gigas* Pacific oyster	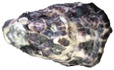	CALCITIC OL: prismatic IL: foliated + chalky lenses	AIM	4.35	0.57%	76.74%
			ASM > 10 kDa	1.14		
			1 < ASM < 10 kDa	0.18		
*Cerastoderma edule* Common cockle	* 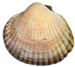 *	ARAGONITIC OL: crossed-lamellar IL: complex crossed-lamellar	AIM	0.51	0.08%	65.25%
			ASM > 10 kDa	0.10		
			1 < ASM < 10 kDa	0.18		
*Venerupis philippinarum* Manila clam	* 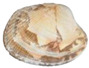 *	ARAGONITIC OL: composite prismatic IL: crossed-lamellar becoming homogeneous	AIM	1.18	0.15%	77.96%
			ASM > 10 kDa	0.22		
			3 < ASM < 10 kDa	0.09		
			1 < ASM < 3 kDa	0.03		
*Venus verrucosa* Warty venus	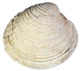	ARAGONITIC OL: composite prismatic ML: crossed-lamellar IL: homogeneous	AIM	1.11	0.20%	55.23%
			ASM > 10 kDa	0.72		
			1 < ASM < 10 kDa	0.18		

* The mineralogy and shell microstructures are indicated in the second column. Abbreviations: OL: outer layer; ML: middle layer; IL: internal layer; AIM: acetic acid-insoluble matrix; ASM: acetic acid-soluble matrix.
